# Thickness of Subcutaneous Fat Is a Predictive Factor of Incisional Surgical Site Infection in Crohn's Disease Surgery: A Retrospective Study

**DOI:** 10.1155/2018/1546075

**Published:** 2018-07-22

**Authors:** Xingchen Cai, Weisong Shen, Zhen Guo, Yi Li, Lei Cao, Jianfeng Gong, Weiming Zhu

**Affiliations:** ^1^Department of General Surgery, Jinling Hospital, Nanjing Medical University, Nanjing, China; ^2^Department of General Surgery, Jinling Hospital, Medical School of Nanjing University, Nanjing, China

## Abstract

**Background:**

Incisional surgical site infection (iSSI) is a frequent postoperative complication of abdominal surgeries in patients with Crohn's disease (CD). In this study, we investigated the association between thickness of subcutaneous fat (TSF) and iSSI in patients with CD undergoing intestinal resections.

**Patients and Methods:**

Patients with CD who had undergone abdominal surgery from January 2014 to January 2017 were included in this retrospective study. Patients' TSF and other possible predictors of iSSI, including clinical characteristics, preoperative medications, hematological index, surgery-related data, and postoperative outcomes, were collected. Univariate and multivariate statistical analyses were used to examine the potential factors. Receiver operating characteristic (ROC) curve analysis was used to evaluate the predictive value of factors.

**Results:**

The patient cohort comprised 246 patients (167 male (67.9%); mean age 35.7 ± 12.4 years; mean disease duration 69.6 ± 60.8 months). The incidence of iSSI was 24.8% (61/246). TSF was a significant predictor of iSSI (OR 1.079, 95% CIs (1.020, 1.142), *P* = 0.008), being 13.7 mm in patients with iSSI and 9.9 mm in those without iSSI (*P* < 0.001). Additionally, C-reactive protein (CRP) concentrations (OR 1.059, *P* = 0.003) were also possible predictors of iSSI, as indicated by both univariate and multivariate analysis. A model of iSSI comprising TSF and CRP concentrations was moderately accurate (AUC 0.827, CIs (0.766, 0.888)).

**Conclusions:**

Preoperative TSF and CRP independently affect iSSI in patients with CD undergoing intestinal resections.

## 1. Introduction

Crohn's disease (CD), a chronic inflammatory bowel disorder, involves the whole digestive tract. In recent years, there has been ongoing development of medical treatments, particularly immunosuppressant and biological agents [1, 2]. However, nearly 60% of patients with CD have to undergo at least one abdominal operation [3].

Surgical site infection (SSI) is the commonest infectious complication after abdominal surgery for colorectal cancer and diverticulitis, the incidence being 2%–5% [4]. Furthermore, it is responsible for the highest proportion of costs of hospital-acquired infections [4]. At 14%–24%, its incidence is much higher in patients with CD who have undergone abdominal surgery than in other patients [5–7]. Incisional SSI (iSSI) is a significant form of SSI. Therefore, identifying risk factors for iSSI could help to prevent this complication of surgery in patients with CD.

A range of risk factors contributes to iSSI, including preoperative malnutrition, obesity, intra-abdominal abscess, preoperative blood glucose concentration, and intraoperative blood transfusions [8]. Although patients with CD tend to have multiple risk factors for iSSI, such as malnutrition and immunosuppressive therapies, there have been few studies of risk factors in patients with CD undergoing surgery.

The thickness of subcutaneous fat (TSF) is a common index of nutritional status [9], and previous studies have reported that TSF is a strong risk factor for iSSI after open gastrectomy for gastric cancer and elective colorectal surgery for colorectal cancer [10, 11]. Because of the characteristics of their disease, TSF differs between patients with CD and those with other diseases. To our knowledge, no studies have investigated the relationship between TSF and rate of the iSSI in patients with CD.

The aim of the current study was therefore to focus on investigating any correlation between TSF and the iSSI in CD patients together with assessing other potential risk factors for iSSI, such as smoking, blood loss, and preoperative albumin and C-reactive protein (CRP) concentrations.

## 2. Materials and Methods

### 2.1. Patients

Data on patients with CD who had undergone intestinal resections from January 2014 to January 2017 in the Inflammatory Bowel Disease Center of Jinling Hospital were retrospectively collected. All study patients had undergone routine complete abdominal CT or MRI scans within the two weeks before surgery. This study was approved by the Jinling Hospital Ethics Committee.

Patients who were to undergo elective surgery received optimal therapy aimed at improving their nutritional status and reducing systematic inflammation for at least 3 weeks prior to surgery. The strategies included nutritional therapy, drainage of abscesses, weaning off steroids for >4 weeks in patients who had been taking > 20 mg prednisolone for >6 weeks, withdrawal of antitumor necrosis factor (TNF) for >3 months, antibiotics, and smoking cessation. Indications for emergency surgery included acute intestinal perforation and massive hemorrhage and prevented implementation of the full range of strategies listed above.

Preoperative nutritional support was provided as enteral nutrition (EN) (Nutrison Fibre, Enteral Nutrition Suspension; Nutricia Company, Amsterdam, the Netherlands) through a nasogastric tube in all patients who could tolerate it. Some patients initially received total parenteral nutrition (TPN) and then gradually transited to EN (Peptisorb Liquid, Enteral Nutrition Suspension; Nutricia Company, Amsterdam, the Netherlands). Patients with abdominal abscesses underwent preoperative percutaneous drainage if possible and antibiotic therapy. All operations were performed by the same surgical group, and perioperative management was in accordance with the principle of enhanced recovery after surgery (ERAS). All patients except for those undergoing emergency surgery were fed glucose and sodium chloride solution with nasogastric tube for 24 hours prior to surgery rather than undergoing preoperative mechanical bowel preparation. Incision protection sleeves were used in laparotomies. Prophylactic antibiotics (a single dose of second-generation cephalosporins) were typically administered 30 minutes preoperatively and by intravenous drip on the first and second postoperative days.

### 2.2. Definitions

Diagnoses of iSSI were made in accordance with the guidelines of the Centers for Disease Control and Prevention, Public Health Service, US Department of Health and Human Services [12]. Incisional SSIs were further subclassified as superficial or deep incisional SSI. Superficial incisional SSI was defined as occurring within 30 days of surgery, infection involving only skin or subcutaneous tissue of an incision, and at least one of the following: (1) purulent drainage; (2) organisms isolated from an aseptically obtained culture of fluid or tissue from the superficial part of an incision; or (3) signs or symptoms of infection, including pain, tenderness, localized swelling, redness, heat, and superficial part of an incision deliberately opened by the surgeon, unless the incision was culture-negative. Deep incisional SSI was defined as infection involving the fascia of the incision and at least one of the following: (1) purulent drainage deep in an incision; (2) spontaneous dehiscence or deliberate opening of a deep incision by a surgeon in a patient with the following signs or symptoms: fever (>38°C) and localized pain or tenderness, unless the site is culture-negative; (3) an abscess or other evidence of infection involving a deep incision is found on direct examination, during reoperation, or by histopathologic or radiologic examination. Diagnoses of an iSSI were made by a surgeon or attending physician.

The area selected for measurement of TSF by preoperative CT or MRI scans was one centimeter to the right of the umbilical plane, and the thickness was defined as the distance between the posterior lines of the dermis and the superficial fascia of musculus rectus abdominis [13]. Measurements were performed by two authors (Cai XC and Shen WS) using the Adobe Photoshop (version 16.0.0.88).

### 2.3. Data Collection

Specific variables recorded for each patient were as follows: age, gender, duration of disease, disease behavior, disease location, perianal disease, intra-abdominal septic complications (IASCs) including anastomotic leak, intra-abdominal abscess, or enterocutaneous fistula, previous abdominal surgery, BMI, smoking habits, preoperative hospitalizations, preoperative nutritional support and drug administration, surgical approach, length and class of incision, duration of operation, intraoperative blood loss, anastomosis position, postoperative IASCs, and stoma creation. Preoperative leukocyte counts and CRP, serum albumin, and blood glucose were all assessed within the 3 days before surgery.

### 2.4. Statistical Analysis

All data were analyzed using the SPSS version 22.0 (SPSS, Chicago, IL, USA). Continuous parametric variables are presented as the mean ± SD, whereas nonparametric data are presented as 5% to 95% confidence intervals. The series mean was chosen to replace missing values. Unpaired *t*-tests were used to compare parametric variables and the Mann–Whitney *U* test for nonparametric data. Categorical data were analyzed by the *χ*^2^ tests and contingency tables. Univariate and multivariate logistic regression analyses were performed to identify possible predictors of postoperative iSSI. Backward Wald was selected to choose confounders, and only variables with *P* < 0.10 in univariate analysis were assessed by multivariate logistic regression analysis. Predictive accuracy was assessed by receiver operating characteristic (ROC) curve analysis. Multi-index combined ROC curve analysis was first performed with multivariate logistic regression analysis and output probabilities of predicted values, after which, probabilities were further assessed by constructing ROC curves. Overall, *P* < 0.05 was considered to denote statistical significance.

## 3. Results

### 3.1. Clinical Characteristics of Patients

The study cohort comprised 246 patients (167 men, 67.9%) of average age 35.7 years (8 to 71 years). The disease duration was 69.6 ± 60.8 months and the average TSF 10.87 mm (range 0.76–37.92 mm). The incidence of iSSI in this cohort was 24.8% (61/246).

Relevant patient data, preoperative medications, and operative data are summarized in Table 1 and hematological indexes in Table 2. TSF was 13.7 mm in patients who developed an iSSI and 9.9 mm in those who did not (*P* < 0.001). Univariate analysis showed that the potential risk factors for iSSI (*P* < 0.1) in patients with CD included previous abdominal surgery, length of incision, type of incision, preoperative CRP, hemoglobin, and blood glucose. To investigate associations between individual risk factors and development of iSSI, the factors were subjected to multivariate analysis, as shown in Table 3. TSF was a significant predictor of iSSI (OR 1.079, 95% CIs (1.020, 1.142), *P* = 0.008). Additionally, preoperative CRP concentrations were much higher in the iSSI group (OR 1.059, CIs (1.019, 1.100), *P* = 0.003).

### 3.2. ROC Curve Analysis

**Figure 1:**
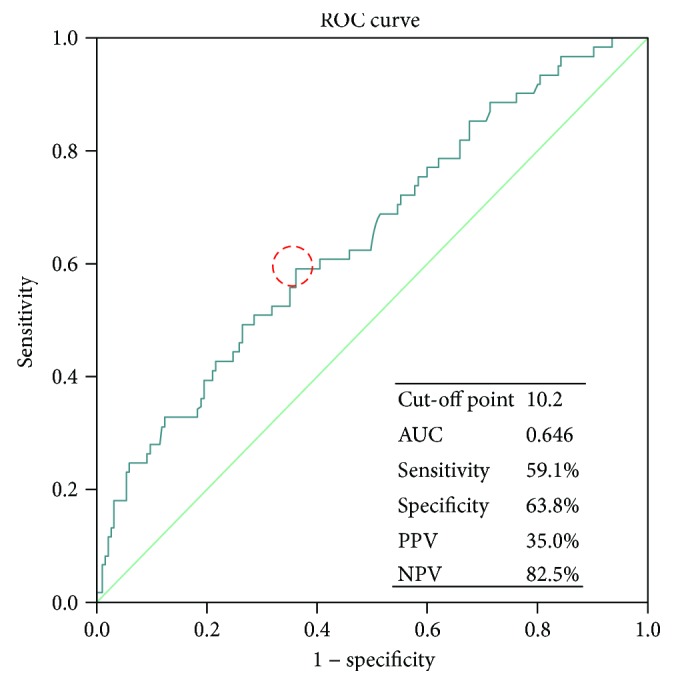
ROC curve showing preoperative TSF predictive value of postoperative iSSI. TSF: thickness of subcutaneous fat; ROC: receiver operating characteristic; AUC: area under the curve; PPV: positive predictive value; NPV: negative predictive value.

**Figure 2:**
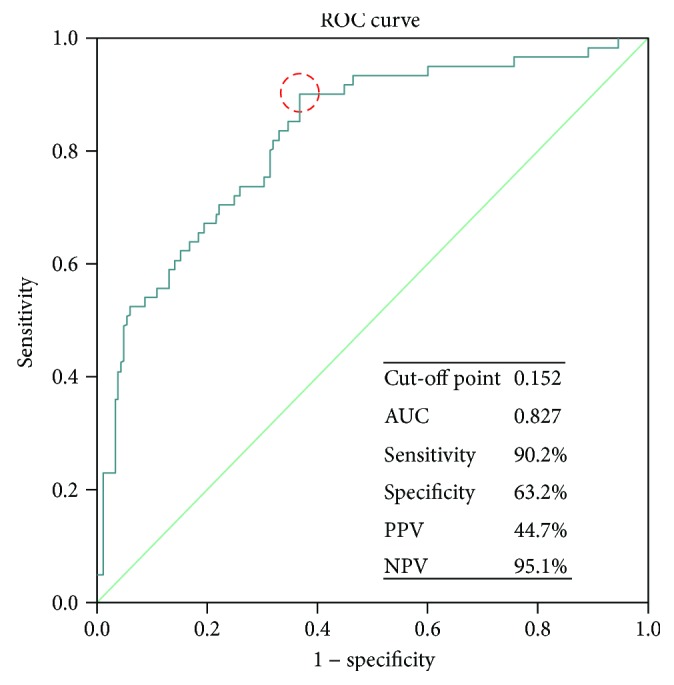
ROC curve showing the model within preoperative TSF and CRP levels predictive value of postoperative iSSI. CRP: C-reactive protein; ROC: receiver operating characteristic; AUC: area under the curve; PPV: positive predictive value; NPV: negative predictive value.

ROC curves were constructed to determine the predictive value of TSF for iSSI in patients with CD (Figure 1). The area under the curve (AUC) was 0.646 (CIs (0.583, 0.706)), the sensitivity of the cut-off value of 10.2 mm being 59.1% and the specificity 63.8%. Furthermore, the negative predictive value (NPV) was 82.5% and the positive predictive value (PPV) only 35.0%. An attempt was made to build a model for iSSI prediction comprising preoperative TSF and CRP concentrations. ROC curves were then constructed to analyze the predictive value, as shown in Figure 2. In this model, the cut-off point was 0.152 and AUC 0.827 (CIs (0.766, 0.888)). Cut-off point 0.152 was corresponding to TSF cut-off value 10.2 mm and CRP cut-off value 8.6 mg/L. The sensitivity was 90.2% and the specificity 63.2%. The NPV of the cut-off point was 95.1% and the PPV 44.7%, indicating that the model has a higher predictive value for iSSI than TSF or CRP alone.

### 3.3. Predictors of iSSI

Preoperative TSF and CRP concentrations were classified according to cut-off values of 10.2 mm and 8.6 mg/L, respectively. Multivariate analysis was performed to identify predictors significantly associated with iSSI; these are presented in Table 4. TSF (OR 2.519, CIs (1.350, 4.698), *P* = 0.004) and CRP (OR 4.556, CIs (2.378, 8.728), *P* < 0.001) were both associated with an increased risk of iSSI.

## 4. Discussion

Surgery for CD is challenging, often being accompanied by multiple postoperative complications. Infective complications are an important type of postoperative complication in these patients, previous studies having mainly focused on exploring risk factors for development of intra-abdominal sepsis [14–16]. Postoperative iSSI, the major form of infective complication, increases hospitalization costs, prolongs hospital stay, and negatively affects patients' quality of life. The incidence of iSSI is reportedly much higher in patients with CD than in those with other diseases [5, 17]; however, there are few studies on iSSI after abdominal surgery for CD. In the present retrospective study of patient data, our primary focus was on the relationship between postoperative iSSI and thickness of abdominal subcutaneous fat in patients with CD. First, the incidence of iSSI in our patients with CD who had undergone intestinal resection was 24.8% (61/246), this rate being quite close to that reported by Bhakta et al. [5]. Second, we found preoperative TSF to be an independent risk factor for iSSI after surgery for CD. Third, preoperative CRP concentrations were also predictors of iSSI, and a model including TSF and CRP concentrations had better predictive value (AUC 0.827) than these predictors alone.

The body fat distribution of patients with CD, especially those with aggressive disease, differs from that of other persons, this being mainly characterized by a higher ratio of visceral to subcutaneous fat [18]. TSF is associated with BMI, and several studies have shown that TSF is an independent risk factor for iSSI after elective colorectal surgeries [11, 19]. A relationship between iSSI and TSF makes intuitive sense and has long been proven. Soper and Bump [20] reported that only patients with depth of subcutaneous fat > 3 cm developed iSSI after undergoing abdominal hysterectomy. Fujii et al. [11] reported a cut-off value for TSF of 20 mm in Asian patients who developed iSSI after undergoing elective colorectal surgery. However, CD is a type of wasting disease, affecting individuals often having very thin abdominal subcutaneous fat, even as thin as a wafer. In our 246 patients, the minimum TSF was only 0.76 mm and the mean 10.87 mm. Therefore, previously demonstrated associations between TSF and iSSI cannot validly be assumed to apply to patients with CD. We found that TSF is an independent risk factor for iSSI in patients undergoing surgery for CD, the cut-off point being 10.2 mm. This cut-off value is markedly lower than the 20 mm reported by Fujii et al., indicating that CD differs from other diseases such that it is inappropriate to mechanically predict postoperative complications in patients using predictive models developed for other diseases.

CRP is synthesized in hepatocytes, and serum concentrations increase rapidly during inflammatory responses [21]; thus, serum CRP concentration often plays a role as a marker of inflammation in a clinical context. Given that peak CRP concentrations occur about 2 days after the initiation of an acute inflammatory response and facilitate detection of such responses, CRP concentrations can be used to predict postoperative infective complications [22]. Serum CRP concentration on the fourth postoperative day can be used to eliminate postoperative infectious complications [23]. Additionally, CRP concentration is reportedly the most frequently used noninvasive biomarker for evaluating the activity of CD. Furthermore, in a previous study, we found that preoperative CRP concentration is a practical predictor of intra-abdominal septic complications after performing primary anastomoses in patients with CD [14]. In the present study, we found that preoperative CRP concentration is a predictor of postoperative iSSI in patients with CD. This finding is in line with our hypothesis that preoperative CRP concentration reflects the activity and severity of CD.

Whether preoperative administration of infliximab influences the incidence of postoperative iSSI remains controversial. Some meta-analyses have indicated that preoperative infliximab is associated with increased risk of infectious complications in patients with CD who undergo surgeries [24, 25]. However, the findings of another meta-analysis suggested that infliximab does not increase the risks of postoperative infective complications [26]. Uchino et al. reported that preoperative administration of infliximab is not a risk factor for iSSI [27]. Thus, the relationship between such medications and postoperative iSSI is still under discussion. In our study, infliximab administration showed no correlation with postoperative iSSI in patients with CD.

The incidence of postoperative IASCs in this cohort was 4.5% (11/246), which is comparable to the 5%–20% reported by previous studies [15, 16, 28]. Only three patients with IASCs required revision surgery, the remaining IASCs resolved with percutaneous drainage. Though postoperative IASCs were strongly associated with iSSI, especially deep iSSI, we considered that IASCs have limited predictive value because they develop later than other indexes. We therefore did not include postoperative IASCs as a predictor of iSSI.

Our study had some limitations. First, it was a single-center retrospective study. However, it was relatively large, including 246 patients. However, our findings are less persuasive than those of a prospective study would be and lack widespread applicability. Second, we found that TSF is a predictor of iSSI; we did not evaluate associations between iSSI severity and amount of TSF. Third, only 16 patients in our series had undergone emergency abdominal operation surgery, which may have influenced the accuracy of our data and the universality of our results. Despite these limitations, our findings indicate that TSF has predictive value for postoperative iSSI in patients with CD.

## 5. Conclusion

This study is the first to investigate the relationship between TSF and iSSI after abdominal surgery for CD. Our results suggest that preoperative TSF and CRP concentrations can predict development of postoperative iSSI. We therefore recommend the use of incision protection supplementary materials, more frequent checking of incisions, and prompt treatment to minimize the incidence of postoperative iSSI in patients with Crohn's disease, preoperative TSF >10.2 mm, and CRP > 8.6 mg/L.

## Figures and Tables

**Table 1:** Clinical and operational characteristics of patients in the research group.

Variable	Incisional SSI		*P* value
	Present (*n* = 61)	Absent (*n* = 185)	
Gender (male/female)	41/20	126/59	0.507
Age (years)	36.5 ± 13.3	35.5 ± 12.7	0.593
Duration of disease (months)	65.6 ± 60.5	70.9 ± 61.1	0.554
Thickness of subcutaneous fat (mm)	13.7 ± 7.7	9.9 ± 6.1	<0.001
Disease behavior, *n* (%)			0.203
Stricture	38 (62.3)	87 (47.0)	—
Penetration	21 (34.4)	91 (49.2)	—
Others	2 (3.3)	7 (3.8)	—
Disease location, *n* (%)			0.373
Ileum	17 (27.9)	65 (35.1)	—
Colon	6 (9.8)	25 (13.5)	—
Ileocolon	38 (62.3)	93 (50.3)	—
Upper digestive tract	0 (0)	2 (1.1)	—
Perianal disease, *n* (%)	15 (24.6)	61 (33.0)	0.219
Abdominal abscess, *n* (%)	10 (16.4)	15 (8.1)	0.063
Previous abdominal surgery for CD, *n* (%)			0.119
0	25 (41.0)	102 (55.1)	—
1	26 (42.7)	65 (35.2)	—
>1	10 (16.3)	18 (9.7)	—
Smoking, *n* (%)	10 (16.3)	15 (8.1)	0.063
Length of preoperative hospitalization (days)	9.7 ± 16.5	8.1 ± 4.6	0.222
BMI (kg/m^2^)	17.4 ± 5.5	16.7 ± 5.0	0.380
Preoperative PN, *n* (%)	13 (21.3)	34 (18.4)	0.613
Preoperative EN, *n* (%)	47 (77.0)	157 (84.9)	0.159
Preoperative drug administration, *n* (%)			
5-ASA	15 (24.6)	59 (31.9)	0.281
Steroids	5 (8.2)	12 (6.5)	0.771
Immunosuppressant	20 (32.8)	60 (32.4)	0.959
Anti-TNF *α* antibody	6 (9.8)	12 (6.5)	0.400
Emergency operation, *n* (%)	5 (8.2)	11 (5.9)	0.553
Surgical approach, *n* (%)			0.117
Laparotomy	52 (85.2)	140 (75.7)	—
Laparoscopic	9 (14.8)	45 (24.3)	—
Anastomosis position, *n* (%)			0.255
Nonanastomosis	11 (18.0)	39 (21.1)	—
Ileal anastomosis	15 (24.6)	62 (33.5)	—
Ileocolostomy	35 (57.4)	84 (45.4)	—
Stoma creation, *n* (%)			0.532
Ileostomy	13 (21.3)	44 (23.8)	—
Colostomy	4 (6.6)	3 (1.7)	—
Length of incision (cm)	12.2 ± 3.5	10.6 ± 4.2	0.009
Incision class, *n* (%)			0.011
Contaminated	51 (83.6)	174 (94.1)	—
Dirty	10 (16.4)	11 (5.9)	—
Duration of operation (min)	148.6 ± 49.5	141.2 ± 52.5	0.337
Intraoperative blood loss (mL)	218.2 ± 157.4	182.5 ± 116.2	0.059
Postoperative transferred to ICU, *n* (%)	10 (16.4)	19 (10.3)	0.189
Postoperative IASCs, *n* (%)			0.020
Anastomotic leak	3 (8.2)	2 (1.1)	—
Intra-abdominal abscess	3 (4.9)	1 (0.5)	—
Enterocutaneous fistula	1 (1.6)	1 (0.5)	—
Reoperation, *n* (%)	2 (3.3)	1 (0.5)	0.153

Data are reported as the number of patients (%) or mean ± SD. SSI: surgical site infection; CD: Crohn's disease; BMI: body mass index; PN: parenteral nutrition; EN: enteral nutrition; IASCs: intra-abdominal septic complications.

**Table 2:** Hematological index.

Variable	Incisional SSI		*P* value
	Present (*n* = 61)	Absent (*n* = 185)	
Preoperative leukocyte (10^9^/L)	6.1 ± 3.9	5.4 ± 2.5	0.154
Preoperative CRP (mg/L)	32.0 ± 44.5	6.7 ± 18.9	<0.001
Preoperative albumin (g/L)	36.7 ± 7.5	37.0 ± 5.7	0.723
Preoperative hemoglobin (g/L)	112.0 ± 20.2	118.3 ± 16.3	0.014
Preoperative blood glucose (mmol/L)	5.8 ± 1.7	5.2 ± 1.2	0.029

Data are reported as mean ± SD. SSI: surgical site infection; CRP: C-reactive protein.

**Table 3:** Multivariable logistic regression analysis of predictors of iSSI in patients with CD after abdominal surgery.

Variable	95% CI	OR	*P* value
Thickness of subcutaneous fat	1.020–1.142	1.079	0.008
Abdominal abscess	0.236–4.500	1.030	0.969
Smoking	0.564–5.116	1.698	0.347
Length of incision	0.938–1.153	1.040	0.458
Incision class	0.113–6.450	0.854	0.878
Blood loss	0.998–1.004	1.001	0.497
Preoperative CRP	1.019–1.100	1.059	0.003
Preoperative hemoglobin	0.975–1.027	1.001	0.953
Preoperative blood glucose	0.924–1.597	1.215	0.164

OR: odds ratio; CI: confidence interval; CD: Crohn's disease; CRP: C-reactive protein; IASCs: intra-abdominal septic complications.

**Table 4:** Multivariable logistic regression analysis of iSSI predictors classified according to cut-off value.

Variable	95% CI	OR	*P* value
Thickness of subcutaneous fat	1.350–4.698	2.519	0.004
Preoperative CRP	2.378–8.728	4.556	<0.001

OR: odds ratio; CI: confidence interval: CRP: C-reactive protein.

## Data Availability

The data used to support the findings of this study are available from the corresponding author upon request.
